# Gut microbiota and quantitative traits divergence at different altitude of long-tailed dwarf hamsters, *Cricetulus longicaudatus*

**DOI:** 10.3389/fmicb.2024.1531629

**Published:** 2025-01-24

**Authors:** Yue Ren, Mengfan Tao, Xiaoliang Wang, Xinsheng Pu, Guangtong Guo, Kuiyou Chen, Bingyu Zhao, Yu Hou, Xin'gen Yang, Yumei Xu

**Affiliations:** ^1^College of Plant Protection, Shanxi Agricultural University, Taiyuan, China; ^2^Pingyao County Forestry Bureau, Jinzhong, China; ^3^Shanxi Key Laboratory of Integrated Pest Management in Agriculture, College of Plant Protection, Shanxi Agricultural University, Taiyuan, Shanxi, China

**Keywords:** *Cricetulus longicaudatus*, gut microbiota, different altitudes, quantitative traits, adaption

## Abstract

To investigate the community structure and diversity of gut microflora and their function in body mass regulation, as well as the effects of various locations on gut microbiota and *Cricetulus longicaudatus* body mass regulation at various elevations. We examined the diversity, abundance, and community structure of the gut microbiota of long-tailed dwarf hamsters from eight regions in Shanxi province during summer using 16S rDNA sequencing technology and analyzed the relationships between these microbiota and environmental variables as well as morphological indicators. The results revealed Firmicutes and Bacteroidetes as the dominant phyla at the phylum level, with Lactobacillus emerging as the predominant genus. We observed differences of gut microflora between different areas, and this diversity is affected by altitude. The high-altitude areas individuals had lower β diversity of gut microbiota than the low-altitude area. Moreover, the body and skull indexes of long-tailed dwarf hamsters also changed with altitude. The result presented in this study indicated that the body size of long-tailed dwarf hamsters conforms to Bergmann's law. And Providencia had significant correlation with body size. Finally, functional analysis of the gut microbiota showed changes in metabolic function that depended on elevation, and collinear network analysis showed how the gut microbiota interacts with each other. All of these results suggest that long-tailed hamsters are different depending on their altitude, with altitude being the main factor affecting both the structure of microbes and the way their metabolism works. This study shows that altitude has a big effect on the gut microbiota and phenotypic traits of long-tailed hamsters. It also shows how well this species can adapt to changes in altitude.

## 1 Introduction

The gut microflora is the densest microbial community of mammals and one of the most diverse (Carvalho et al., 2012). Mammalian gut microflora colonize the body from birth and develop into relatively stable microbial communities as the host grows, which are finely coordinated with the host's physiology and co-evolve with the host (Hooper et al., 2002; Bäckhed et al., 2004). The gut microflora plays an indispensable role in the host organism. Researchers have shown that gut microflora affects the immune systems of mammals by making certain chemicals, like short-chain fatty acids, that change how immune cells work. These metabolites support tissue growth and repair, strengthen defenses against external infections, and preserve the host's physiological health (Wang et al., 2010; Liu et al., 2012, 2021). Gut microbiota can help the host maintain vital physiological processes even in harsh conditions by improving food digestion and nutrient absorption efficiency, maximizing energy conversion rates within the host organism, and strengthening the host's natural resilience (Tsuchida et al., 2004). The gut microbiota of mammals is affected by many factors, with altitude being the primary determinant that can impact the composition and diversity of the gut microbiota (Hao et al., 2024). For example, previous research has demonstrated a significant influence of altitude variations on the composition of the gut microbiota in donkeys (*Equus africanus asinus*; Guo et al., 2022), European mouflon sheep (*Ovis orientalis musimon*), and blue sheep (*Pseudois nayaur*; Sun et al., 2019). Specifically, animals that live at lower elevations have significantly more diverse and rich gut microbial communities than animals that live at higher elevations. The diversity of gut microbiota and colonization patterns are significantly influenced by altitude, according to studies on *Mus musculus* (Suzuki et al., 2019), *Ochotona curzoniae* (Li et al., 2016), *Chiroptera* spp. (Phillips et al., 2012), and European *Lampyridae* (Sudakaran et al., 2012). In all of these studies, there was a significant correlation between differences in altitude and variations in gut microbial composition. The gut microbiota plays a crucial role in facilitating animals' adaptation to diverse altitudes, thereby enhancing our understanding of their altitude-specific adaptability. Consequently, investigating the impact of altitude factors on the gut microbiota remains an immensely significant study. Each study found a significant correlation between variations in the combination of gut microbes and altitude. In order to better understand animals' altitude-specific adaptability, the gut microbiota is essential in helping them adapt to a variety of altitudes. Thus, examining how altitude influences the gut microbiota is still a crucial research topic.

In addition to affecting the dynamics of animal gut microbiota, elevation variations have a significant effect on animal populations' adaptive strategies, resulting in the evolution of varied survival strategies within the same species at various elevations (Poblete et al., 2018). The gut microbiota is a major factor in the physiological, behavioral, and ecological adaptations that allow animals to survive at varying elevations. For instance, the respiratory and digestive systems of species that live on plateaus, such as the Tibetan antelope (*Pantholops hodgsonii*) and yak (*Bos grunniens*), have experienced significant evolutionary alterations (Ma et al., 2019; Ayalew et al., 2021; Gao et al., 2022). Gut microbiota evolve together with the host and are essential for immunity, digestion, and metabolism, which is why herbivores need effective systems for energy extraction and absorption. While migratory avifauna exhibit altitudinal migration patterns in response to shifting altitude conditions throughout various seasons (Williamson and Witt, 2021), the plateau pika (*Ochotona curzoniae*) uses gut microbiota regulation to lower metabolic rate and enter hibernation during the winter for energy conservation (Wang et al., 2006). Even among closely related species, behavioral, morphological, or physiological variations may be necessary adaptations for survival at varying elevations (Hoffmann and Sgrò, 2011). Studies have shown that changes in the composition of gut microbiota are connected with the physiological state of mice, including their appearance, and alter animal hair color via modulating nutrition absorption, metabolite synthesis, and immune system regulation (Jones, 2016; Li et al., 2022). Variations in altitude also affect mice' physical traits, such as body size and hair color. For example, *Eothenomys miletus* exhibits alterations in body size corresponding to changes in altitude environments (Ren et al., 2023). Investigations on chacarus, *Ochotona curzoniae*, and *O. iliensis* have revealed that hair pigmentation tends to be darker at higher altitudes compared to lower altitudes (Sumner and Swarth, 1924; Conley, 1970). Diverse altitudes may also induce variations in the types and quantities of food, thereby influencing changes in gut microbiota. Our previous research on the long-tailed dwarf hamsters has demonstrated that the feeding environment has a major effect on the diversity and community composition of gut microbiota, which is crucial for controlling animal nutrient absorption and preserving metabolic homeostasis (Cao et al., 2024). Although small mammals, especially rodents, are remarkably tolerant and adaptive, they have a difficult time surviving and reproducing in hostile environments, particularly when they experience altitude changes, and their reactions to environmental changes vary greatly (Guzzetta et al., 2017; Rabiee et al., 2018).

The long-tailed dwarf hamster, *Cricetulus longicaudatus* (Rodentia: Cricetidae), is the most common species in Shanxi Province's countryside and primarily occurs in eastern, central, and western China (Poplavskaya et al., 2018; Yang et al., 2021). Shanxi Province is a typical loess-covered mountain plain with unusually high and low topography and a variety of complex geomorphological kinds. The most emblematic of native animals in this area is the long-tailed dwarf hamster, which is distinguished by its wide range and exceptional adaptability. However, there are still questions about how habitat and altitude affect the gut microbiota composition of this species and how important gut bacteria are for adaptation to various altitudinal habitats. In this study, we examined the impact of varying altitudes on intestinal microbes and physiological morphological changes in long-tailed dwarf hamsters in Shanxi Province using 16S rDNA sequencing technology in conjunction with relevant morphological indicators. The results of this study provided important light on how the gut microbiota contributes to wild rodents' adaptive strategies at different elevations. We hypothesized that altitude might impact the gut microbial diversity of long-tailed dwarf hamsters. We hypothesized that their morphology might be influenced by the structure of their gut microbiota and that there would be variations in gut microbial diversity at different elevations.

## 2 Materials and methods

### 2.1 Animals and experimental design

**Figure 1:**
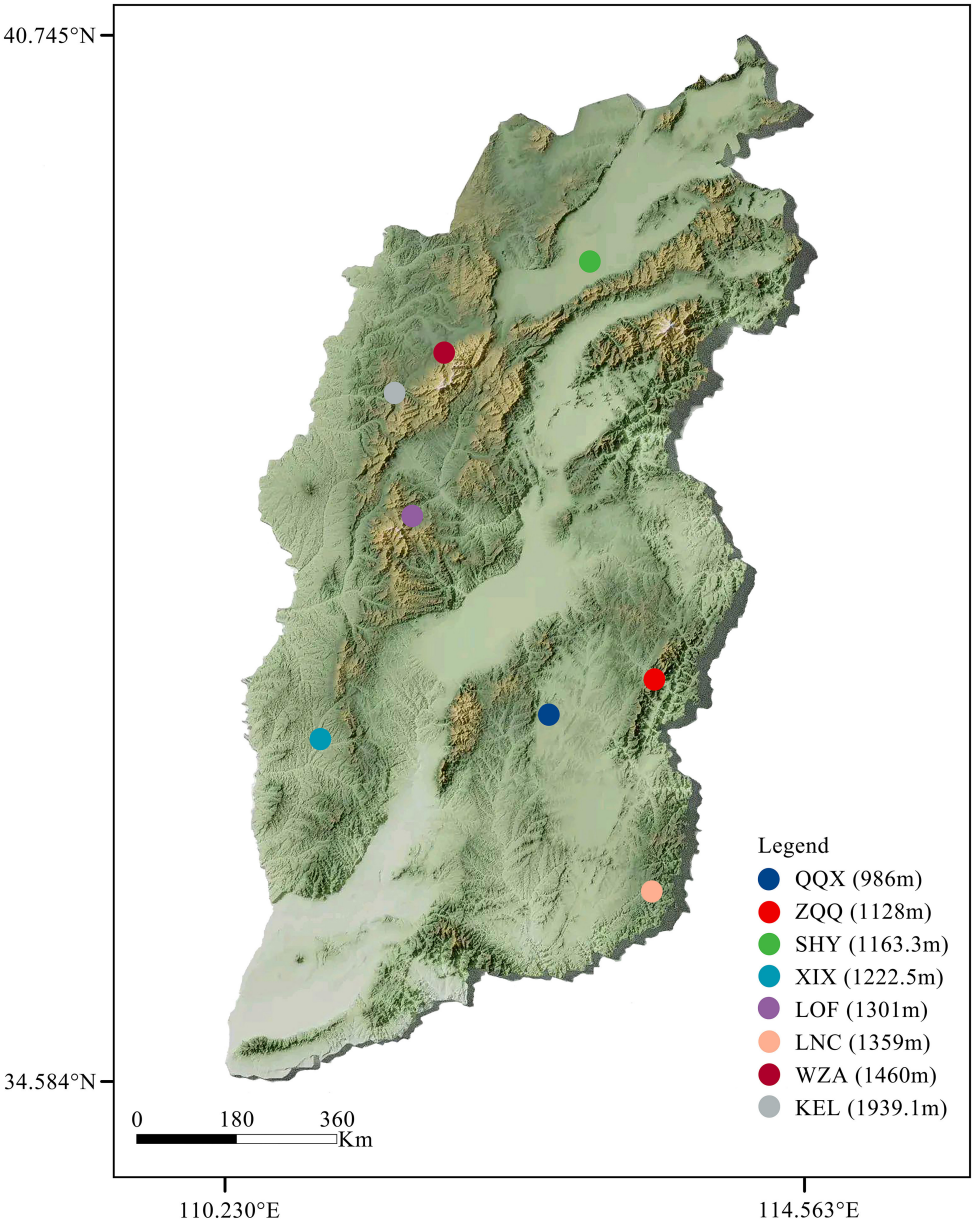
Sampling information of *Cricetulus longicaudatus* used in this study.

The long-tailed dwarf hamster individuals were captured from Qin County (QQX, 112.66°E, 36.91°N), Zuoquan County (ZQQ, 113.29°E, 37.09°N), Shanyin County (SHY, 112.78°E, 39.54°N), Xi County (XIX, 112.78°E, 39.54°N), Loufan County (LOF, 111.79°E, 36.73°N), Linchuan County (LNC, 113.31°E, 35.79°N), Wuzhai County (WZA, 111.62°E, 38.93°N), and Kelan County (KEL, 111.77°E, 38.67°N) in Shanxi Province, during the summer of 2024. The information of the sample site is showed the Figure 1 and Table 1. The animals used in this study were all healthy adult male individuals at the non-breeding stage. We determined the morphological indicators after capture. The determination references were as follows (Yang et al., 2005; Xia et al., 2006): body weight (BW; accurate to 0.01 g), body length (BL), tail length, left forelimb length [FLL(left)], left hindlimb length [HLL(left)], cranial length (CL), cranial basal length (CBL), neurocranium width (NW), cranial height (CH), upper tooth row length on the left side [UTRL(left)], and lower tooth row length on the left side [LTRL(left)] (accurate to 0.01 cm). Samples of rectal feces were taken from long-tailed dwarf hamsters in the wild and then kept at −80°C until they were subjected to high-throughput 16S rDNA sequencing. The experimental protocols were carried out strictly in compliance with the People's Republic of China's Ministry of Science and Technology's Regulations on the Administration of Laboratory Animals (2017 revision).

**Table 1:** Geographical distribution and main environmental conditions in eight regions of the *Cricetulus longicaudatus*.

**Sample site**	**Region**	**Sample number**	**Longitude and latitude**	**Altitude (m)**	**Mean summer temperature (°C)**	**Mean summer humidity (%)**	**Mean summer wind_speed (km/h)**	**Type of vegetation**
Qin County	QQX	6	112.66°E, 36.91°N	986	26	78	8	Three crop two annual
Zuoquan County	ZQQ	8	113.29°E, 37.09°N	1128	28	77	8.6	Warm-temperate evergreen coniferous forest
Shanyin County	SHY	7	112.78°E, 39.54°N	1163.3	27	63	10.3	One crop per annual
Shanyin County	XIX	6	112.78°E, 39.54°N	1222.5	22	68	9.7	One crop per annual
Loufan County	LOF	7	112.78°E, 39.54°N	1301	28	61	8.8	One crop per annual
Linchuan County	LNC	7	113.31°E, 35.79°N	1359.2	31	83	9	Perennial forbs community
Wuzhai County	WZA	7	113.31°E, 35.79°N	1460	20	66	9.8	One crop per annual
Kelan County	KEL	6	111.77°E, 38.67°N	1939.1	25	62	34	One crop per annual

### 2.2 16S rDNA sequencing of gut microbiota and data analyses

Fifty-four long-tailed dwarf hamster samples were subjected to DNA extraction using the E.Z.N.A™ Mag-Bind Soil DNA Kit, and PCR amplification was performed using universal primers. A Qubit 3.0 fluorescent quantitative analyzer was used to measure the library concentration, and 2% agarose gel electrophoresis was used to calculate the library size. All of the samples were then mixed together in a 1:1 ratio. Hieff NGSTM DNA Selection Beads were used to clean up the amplicon product's free primers and primer dimer species. Sangon BioTech received the samples and used a universal Illumina adapter and index to build a library. We used a Qubit^®^ 4.0 Green double-stranded DNA assay to measure the DNA concentration of each PCR product prior to sequencing, and a bioanalyzer to ensure quality control. After sequencing, the RDP database was used to taxonomically classify the representative sequences of bacterial OTU.

We quantified the diversity indices, including the Chao1 and Shannon indices, using OTU richness. We used the ANOVA test for multiple group comparisons and the *T* test for within-sample (alpha) diversity calculation to assess the diversity of the sample's microbial population. Beta diversity is often used with dimensional reduction methods such as principal coordinate analysis (PCoA) or constrained principal component analysis (PCA) to make visual representations. We used the R vegan package (version 2.5-6) to show these analyses, culminating in a final display of scatterplots representing the inter-sample distances. We use LefSe (version 1.1.0) for difference comparison to identify characteristics whose abundances noticeably differ between groups.

### 2.3 Statistic analysis

We used the Mean ± standard deviation to represent the experimental outcomes. One-way ANOVA analysis was used with SPSS 26.0 software to determine the relative abundance, Alpha diversity, and morphological differences of samples between groups. The related dendrogram was created using the ape package, and the hierarchical cluster tree plot was created using R 3.2.6′s hclust function. The distance matrix was subjected to hierarchical clustering analysis in order to create the tree structure, which allowed for the visual inspection of interrelationships. To create a correlation heat map that shows the connections between environmental factors, morphological indicators, and gut microbiota, the Pearson analysis in SPSS 26.0 was utilized. Furthermore, Canoco 5.0 software conducted a redundancy analysis (RDA) to investigate the relationships between dominating genera, morphology, and environmental factors. The data were further examined and network analysis was produced using R 3.2.6 and Gephi v.0.9.2 software (*p* < 0.05, |R| > 0.6). ^*^p < 0.05, ^**^p < 0.01, ^***^p < 0.001 were considered statistically significant.

## 3 Results

### 3.1 Composition of gut microbiota at different elevations

**Figure 2:**
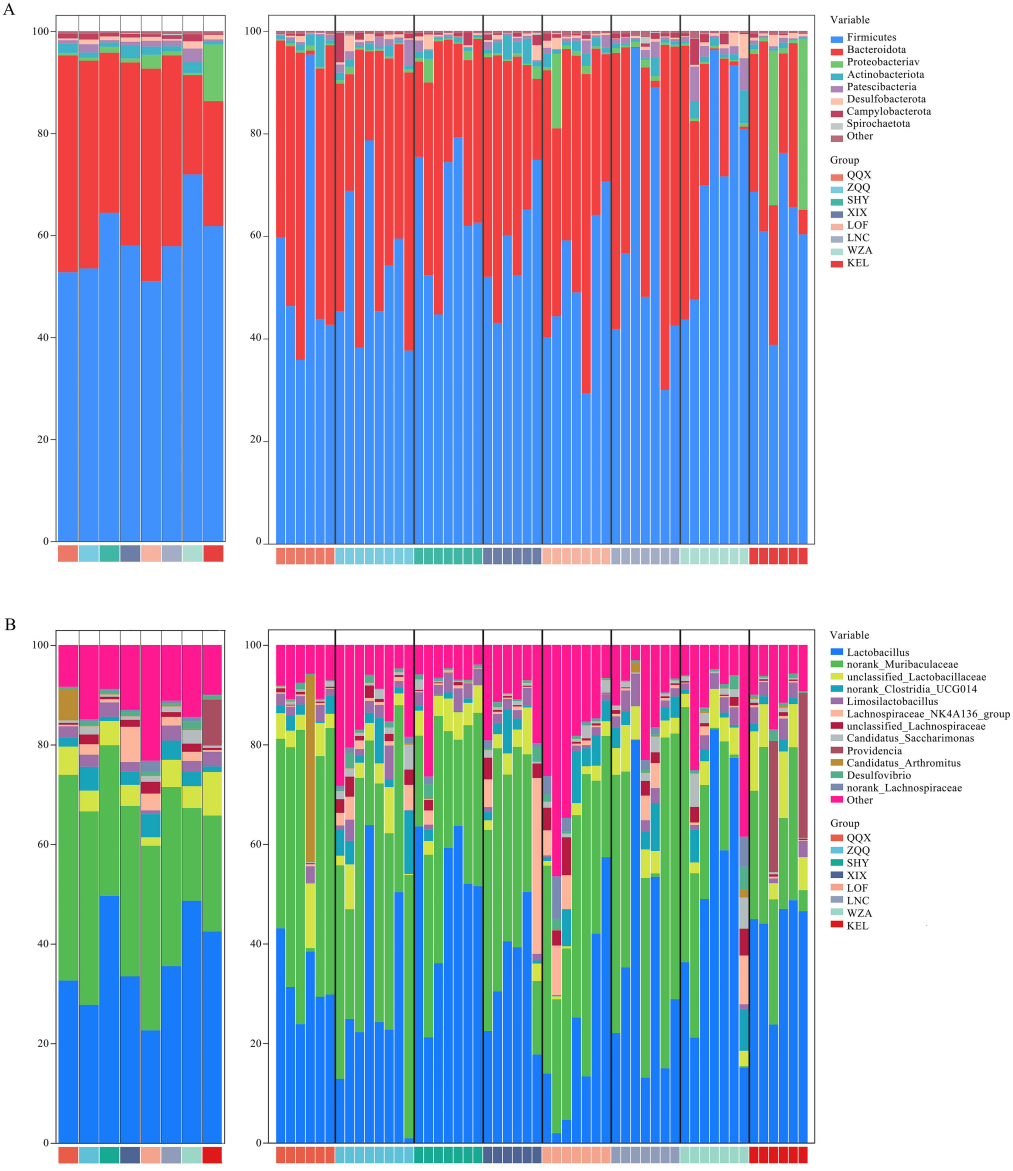
Composition of intestinal microbes in *Cricetulus longicaudatus*. **(A)** Phylum level. **(B)** Genus level.

In the present study, a total of 6,300,654 valid sequences were acquired from the collection of 54 fecal samples from long-tailed dwarf hamsters for 16S rDNA sequencing. After the sequences underwent cluster analysis, 13,769 operational taxonomic units (OTUs) were found, with a 97% similarity criterion. We analyzed the gut microbiota composition of long-tailed dwarf hamsters at the phylum and genus levels based on the OTU classification results. And the results shown that Bacteroidota and Firmicutes dominated all altitude regions at the phylum level (Figure 2A). At the genus level, Lactobacillus, norank_Muribaculaceae, and unclassified_Lactobacillaceae were the dominant genera in the gut microbiota of the long-tailed dwarf hamsters in the eight regions (Figure 2B).

Moreover, our results showed that there were significant variations in microbiota abundance among the eight regions, and these differences varied with altitude. First, the relative abundances of Firmicutes (*p* < 0.05), Proteobacteria (*p* < 0.05), Desulfobacterota (*p* < 0.05), and Spirochaetota (*p* < 0.05) were significantly higher in the long-tailed dwarf hamsters that lived at comparatively higher elevations. On the other hand, the relative abundance of Bacteroidota significantly decreased (*p* < 0.05). Actinobacteriota (*p* < 0.05) and Patescibacteria (*p* < 0.05), on the other hand, demonstrated a considerable rise in relative abundance at higher altitudes, followed by a discernible drop when the height surpassed 1,301 m (Figure 2A). Furthermore, compared to other regions, high altitude areas had considerably greater relative abundances of unclassified Lactobacillaceae (*p* < 0.05), Candidatus Saccharimonas (*p* < 0.05), Providencia (*p* < 0.001), and Desulfovibrio (*p* < 0.05). In contrast, there was a notable decline in the relative abundance of norank_Muribaculaceae (*p* < 0.05), norank_Clostridia_UCG-014 (*p* < 0.05), and Candidatus_Arthromitus (*p* < 0.05). Lactobacillus (*p* < 0.05) and Limosilactobacillus (*P* < 0.01) microbiota relative abundances increased considerably with altitude before dramatically decreasing at 1,301 m above sea level. However, as altitude went up, the relative abundance of Lachnospiraceae_NK4A136_group (*p* = 0.026) and norank_Lachnospiraceae in the microbiota went down a lot, but it went up a lot in WZA argion (Figure 2B).

### 3.2 Diversity of gut microbes at various elevations

**Figure 3:**
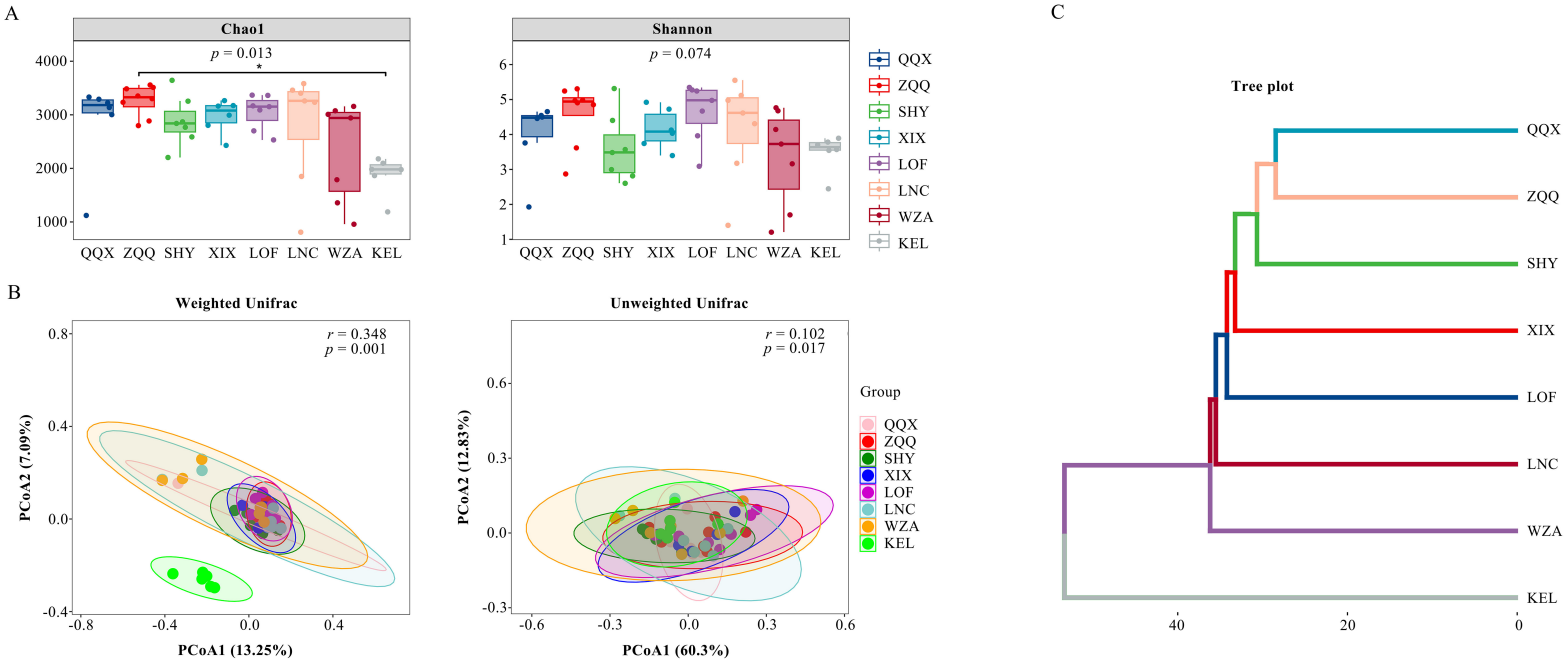
Effects of different altitudes on intestinal microbial diversity of *Cricetulus longicaudatus*. **(A)** Chao 1 and Shannon index for richness, *P < 0.05. **(B)** Principal coordinate analysis (PCoA) of the gut microbial compositions of the *Cricetulus longicaudatus*. **(C)** Cluster analysis of different elevations.

For the gut microbiota of long-tailed dwarf hamsters at each altitude, Figure 3A presents the findings of alpha diversity analysis (Chao1 and Shannon). The Chao1 index only revealed a significant difference between ZQQ and KEL (*p* < 0.05), while no significant difference was found in the other locations (*p* > 0.05). According to the results, the gut microbial of ZQQ individuals was noticeably greater than that of the QQX, SHY, XIX, LOF, LNC, and WZA individuals. The KEL individuals, on the other hand, had a noticeably lower decrease in variety than these six groups. There was no significant variation in the gut microbiota's homogeneity throughout the eight locations, as indicated by the gut microbiota's Shannon index (*p* > 0.05). The unweighted unifrac PCoA results revealed no noticeable variations in the β-diversity of gut microbiota across groups (Figure 3B). Weighted unifrac PCoA analysis of the gut microbiota's β-diversity at various elevations revealed a significant difference between the KEL group and the other seven locations (*p* < 0.01, Figure 3B). The cluster analysis results similarly showed a clear separation of KEL animals from the other groups, which is in line with the weighted unifrac PCoA results (Figure 3C).

**Figure 4:**
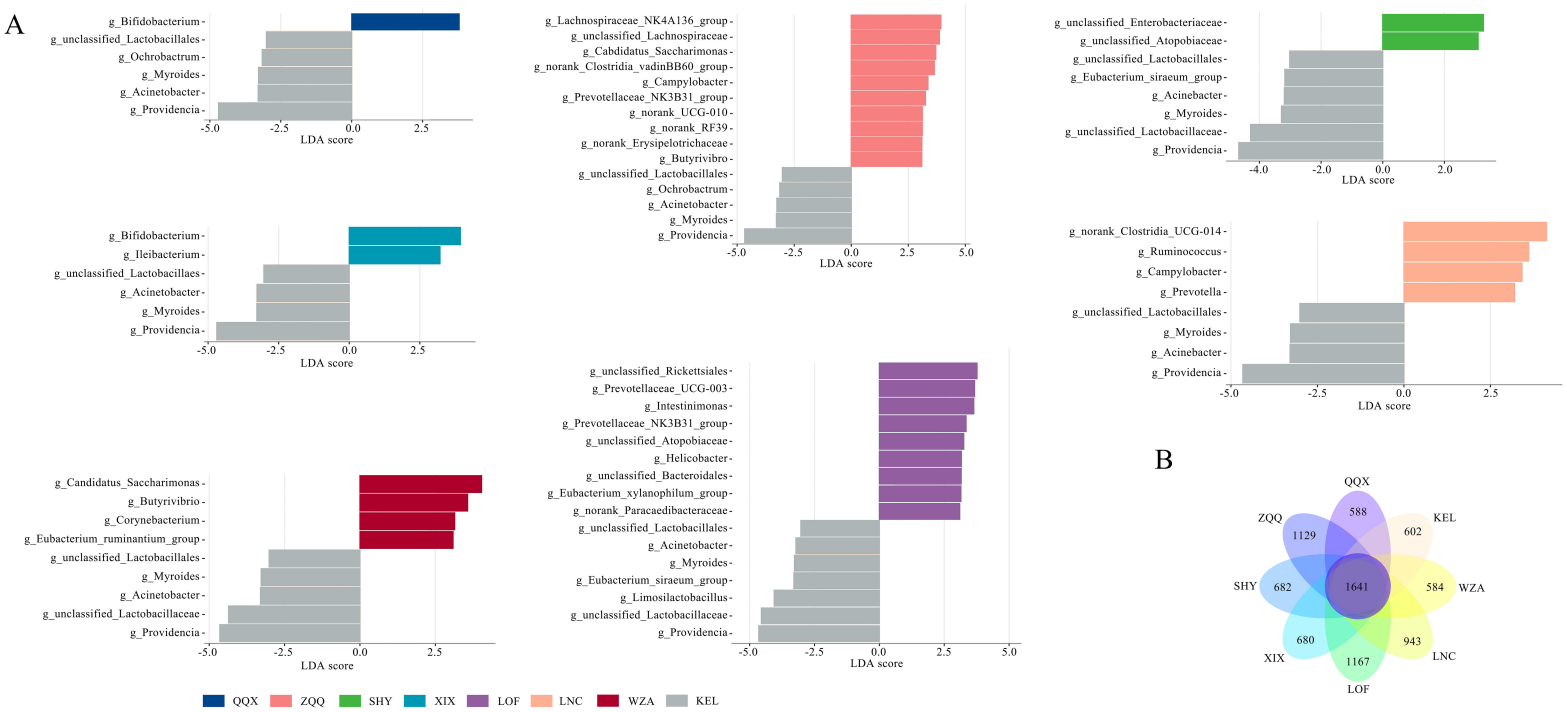
Analysis of gut microbiome difference of *Cricetulus longicaudatus* at different altitudes. **(A)** At genus level, Lefse analysis of intestinal microorganisms in *Cricetulus longicaudatus*. **(B)** Venn diagram of fecal microbials of *Cricetulus longicaudatus* in different regions.

Significant differences in enriched microbial communities between various regions in comparison to KEL were shown by the Lefse analysis, which was used to examine the differential microbial composition between KEL and the other seven regions (Figure 4A). Compared with the KEL region, different regions had different colonies that were enriched significantly. The QQX group had a considerably higher concentration of Bifidobacterium, while the ZQQ group had a significantly higher concentration of Lachnospiraceae_NK4A136_group, unclassified_Lachnospiraceae, and Candidatus_Sacchairmonas. Bifidobacterium and Ileibacterium were highly abundant in the XIX group, while unclassified_Enterobacteriaceae and unclassified_Atopobiaceae were significantly enriched in the SHY group. Unclassified_Rickettsiales and Prevotellaceae_UCG-003 and norank_Clostridia_UCG-014 were among the bacteria that were considerably enriched in the LOF group, while Ruminococcus and Campylobacter were highly enriched in the LNC group. The WZA group had considerably higher levels of Corynebacterium, Butyrivibrio, Eubacterium_ruminantium_group, and Candidiatus_Sacchairmonas. The KEL group had a considerably higher abundance of microbial species, such as Providencia, unclassified_Lactobacillaceae, Myroides, Acinetobacter, Limosilactobacillus, Campylobacter, Ochrobacter, and Eubacterium_siraeum_group, than the other seven categories. The distribution and quantity of the gut microbiota bacteria within each category are directly displayed in the Venn diagram (Figure 4B). Further investigation showed that, among long-tailed dwarf hamsters, the ZQQ and LOF groups had the highest counts of intestinal bacteria, coming in at 1129 and 1167, respectively. The LNC, SHY, XIX, KEL, and QQX groups included 943, 682, 680, 602, and 588 species, whereas the WZA group contained 584 species.

### 3.3 The relationship between environmental factors and gut microbes

**Figure 5:**
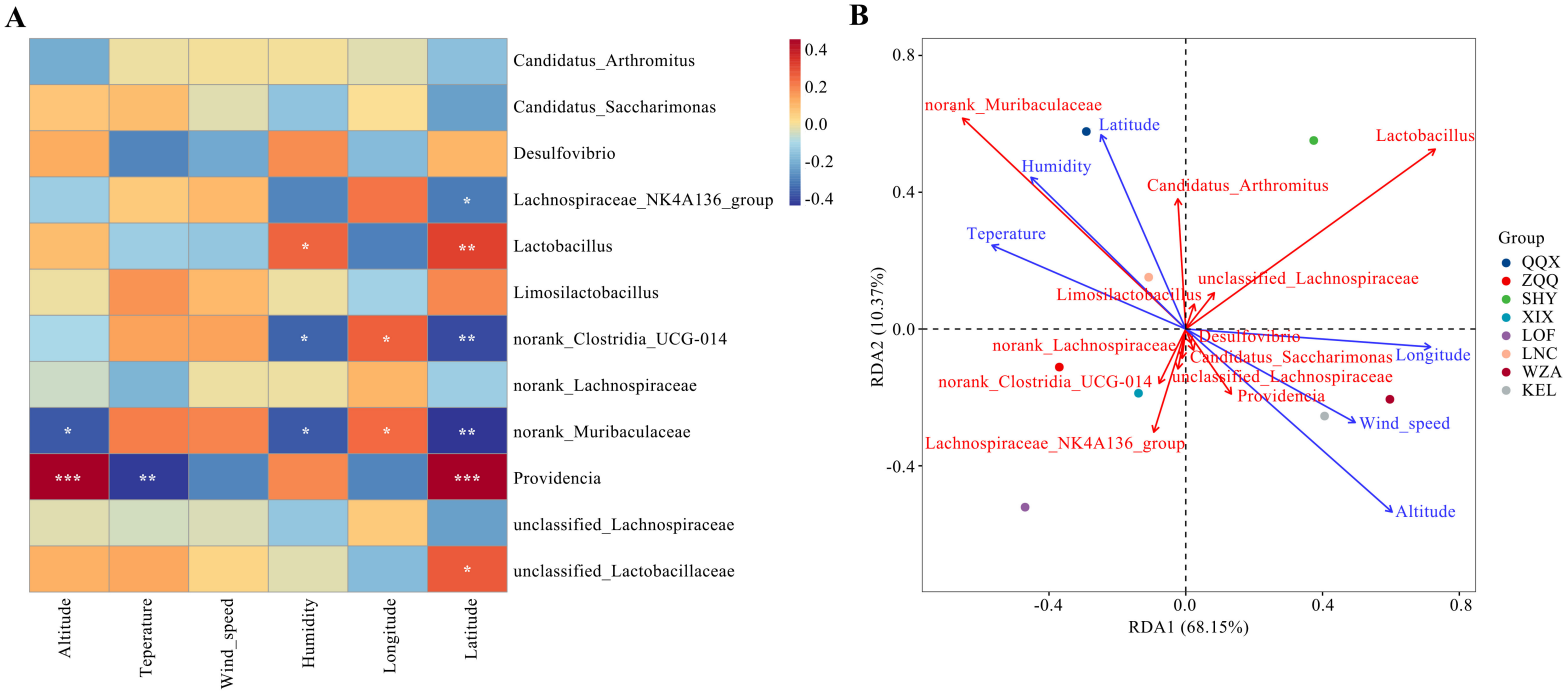
Relationship between environmental factors at different altitudes and intestinal microorganisms in *Cricetulus longicaudatus*. **(A)** Association of environmental factors with gut microbiome Correlation Heatmap. **(B)** Redundancy analyses (RDA) of the correlation between environmental factors and dominant microbial communities. *p < 0.05, **p < 0.01, and ***p < 0.001 were considered statistically significant.

Figure 5A shows the relationship between each bacterial community and environmental variables such as temperature, humidity, wind speed, longitude, altitude, and dimensionality. The results indicate the makeup of different bacterial communities is significantly influenced by environmental conditions. First, in accordance with the results displayed in Figure 5A, altitude exhibited a substantial negative connection with norank_Muribaculaceae (*p* < 0.05) and a positive correlation with Providencia (*p* < 0.001). On the other hand, there was a substantial negative connection (*p* < 0.01) between humidity and providencia. There was a substantial negative connection with norank_Muribaculaceae and norank_Clostridia_UCG-014 (*p* < 0.05) and a significant positive correlation with Lactobacillus (*p* < 0.05) with longitude. However, norank_Muribaculaceae and norank_Clostridia_UCG-014 showed a significant correlation with temperature (*p* < 0.05). Unclassified_Lactobacillaceae (*p* < 0.05), Providencia (*p* < 0.001), and Lactobacillus (*p* < 0.01) showed a substantial positive connection with wind_speed. Conversely, it showed a negative connection (*p* < 0.01) with norank_Clostridia_UCG-014 and norank_Muribaculaceae. Redundancy analysis, which looked at the connection between different environmental conditions and the dominating gut microbiota of long-tailed dwarf hamsters (Figure 5B), further highlighted the effect of altitude on the microbiota. The most significant environmental element influencing changes in the microbiota's composition was found to be altitude, which was followed by longitude and dimension. However, wind speed had little effect on these microbial differences.

### 3.4 The relationship between environmental factors, morphological indices, and gut microbial at different altitudes

**Figure 6:**
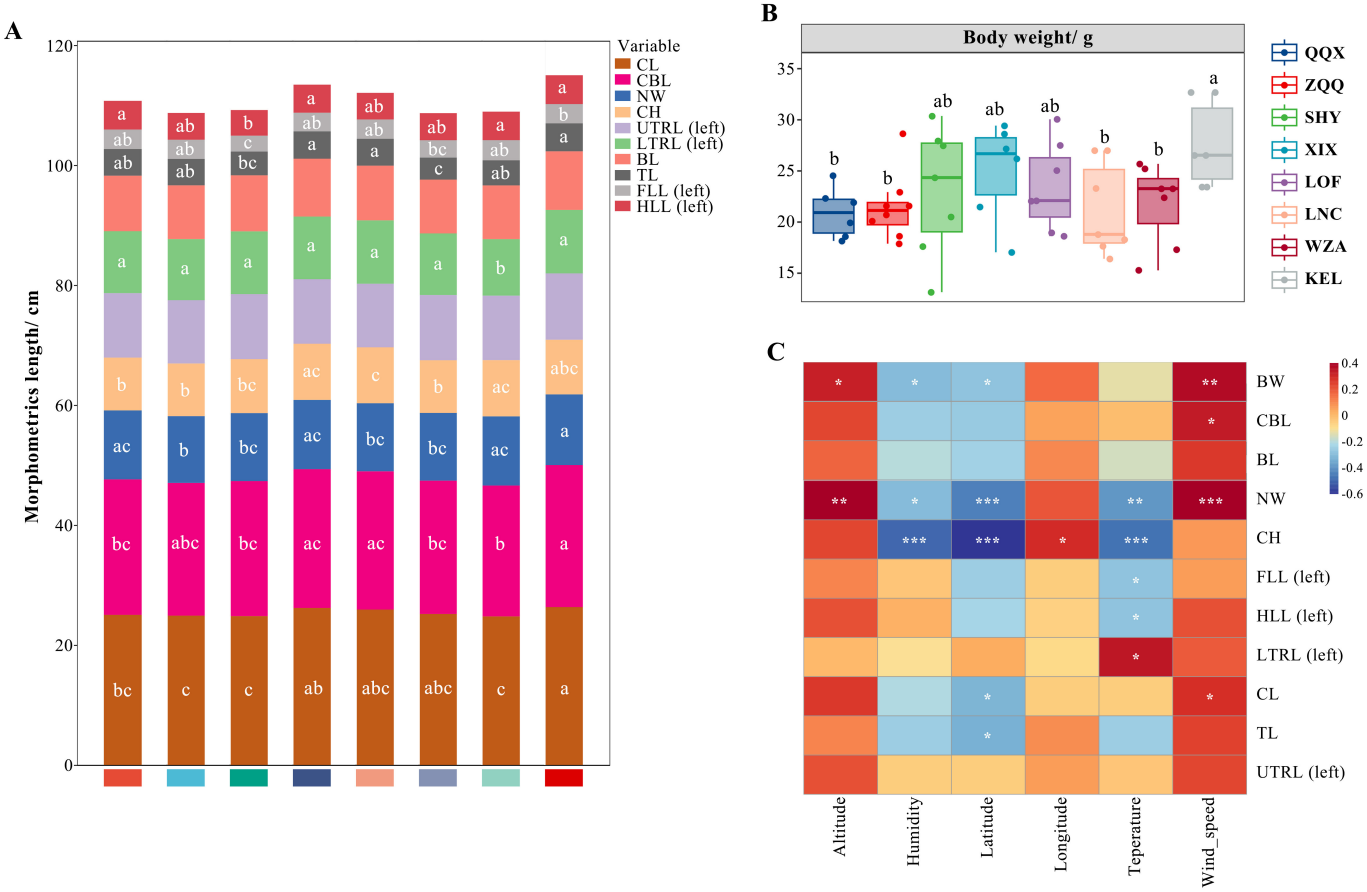
Relationship between phenotypic characteristics and environmental factors in *Cricetulus longicaudatus*. CL, cranial length; CBL, cranial basal length; NW, neurocranium width; CH, cranial height; UTRL (left), upper tooth length (left); LTRL (left), lower tooth row length (left); BL, body length; TL, tail length; FLL (left), left fore limb length; LHLL, left hind limb long; BW, body weight. **(A)** Stacking map of morphological traits of *Cricetulus longicaudatus* at different elevations. **(B)** Body weight of *Cricetulus longicaudatus* at different altitudes. **(C)** Association of environmental factors with phenotypic characteristics Correlation Heatmap. *p < 0.05, **p < 0.01, and ***p < 0.001 were considered statistically significant. Significant group differences were indicated by different alphabetic letters.

A one-way ANOVA (Figures 6A, B) revealed significant variations in body and cranium indices among long-tailed dwarf hamsters at varying elevations. The body weight and body length of KEL individuals were considerably greater than those of the QQX, ZQQ, LNC, and WZA individuals (*F* = 1.715, *p* < 0.05). Furthermore, compared to the other groups, the KEL individuals had a substantially greater cranial length (*F* = 2.192, *p* < 0.05) than the other groups, and had a larger lower tooth row length (left) than the WZA individuals (*F* = 4.848, *p* < 0.05), and its neurocranium breadth was significantly higher than the ZQQ, SHY, LOF, and LNC individuals (*F* = 4.254, *p* < 0.05). Additionally, the left fore and left hind limb length indexes in KEL individuals were substantially larger than those in the SHY group (*F* = 3.594, *p* < 0.05), and the tail length index in KEL individuals was significantly higher than that in the SHY and LNC groups (*F* = 3.492, *p* < 0.05). The KEL individuals' cranial height did not, however, differ significantly from those of the other groups. The phenotypic traits of long-tailed dwarf hamsters and their corresponding environmental conditions were shown to be significantly correlated, according to the correlation study between phenotype and environmental components (Figure 6C). First, there was a substantial positive correlation (*p* < 0.05) between altitude and both body weight and neurocranium breadth. Second, a significant negative correlation was found between humidity and body weight (*p* < 0.05), cranial height (*p* < 0.001), and neurocranial breadth (*p* < 0.05). Additionally, there was a substantial negative correlation between latitude and body weight (*p* < 0.05), cranial height (*p* < 0.001), and neurocranial breadth (*p* < 0.001). The lower tooth row length (left) also exhibited a positive correlation with temperature, as did the left forelimb length (*p* < 0.05), left hind limb length (*p* < 0.05), cranial height (*p* < 0.001), and neurocranial breadth (*p* < 0.05). Additionally, there were positive relationships between wind_speed and cranial length (*p* < 0.05), neurocranial breadth (*p* < 0.001), cranial base length (*p* < 0.05), and body weight (*p* < 0.01).

**Figure 7:**
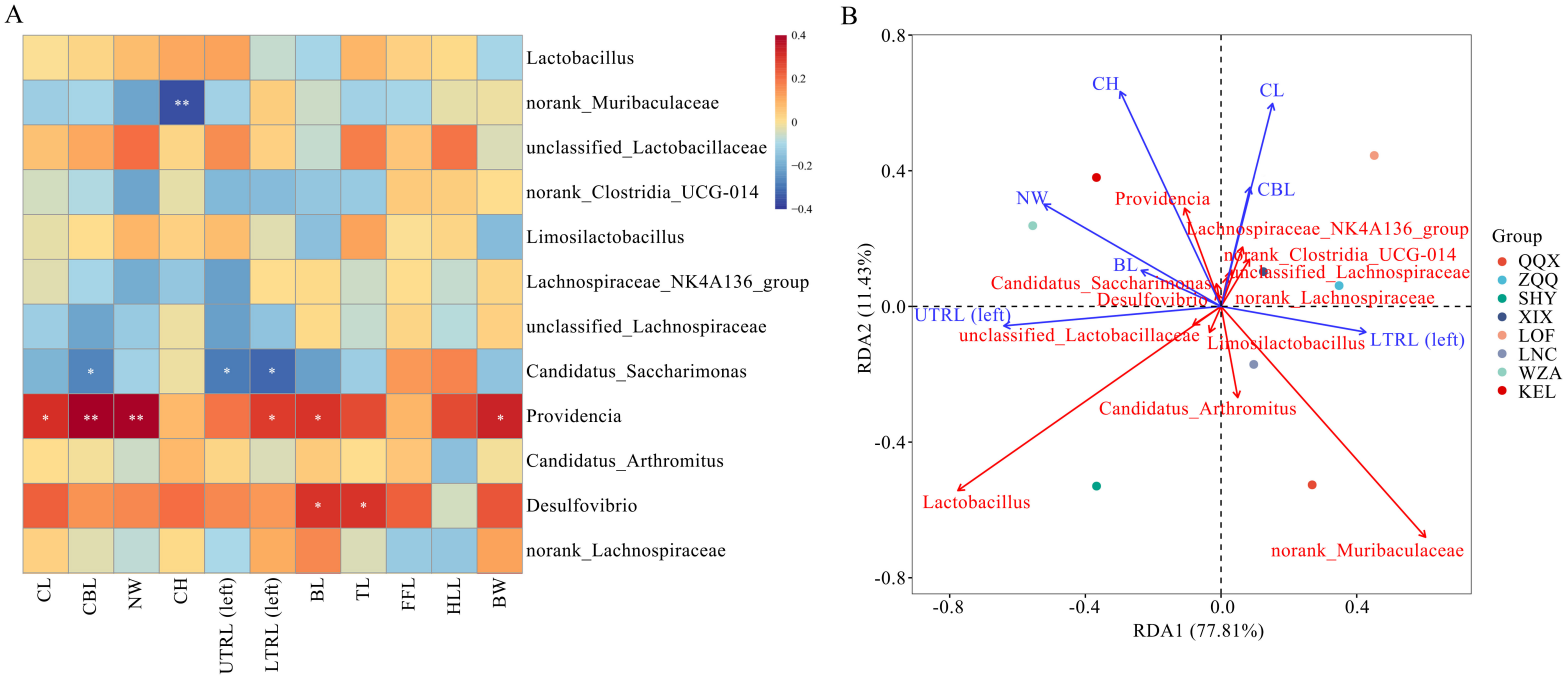
Relationship between phenotypic characteristics at different altitudes and gut microbiome in *Cricetulus longicaudatus*. **(A)** Association of phenotypic characteristics with gut microbiome Correlation Heatmap. **(B)** Redundancy analyses (RDA) of the correlation between phenotypic characteristics and dominant microbial communities. *p < 0.05, and **p < 0.01 were considered statistically significant.

The morphological indicators of long-tailed dwarf hamsters and the relative abundance of dominant bacterial genera in the gut microbiota (the top 12 genera in relative abundance for all samples; Figure 7A) showed that Providencia was significantly positively correlated with the cranial length, cranial base length, neurocranial width, upper tooth length (left), tail length, and body weight of the hamsters (*p* < 0.01); Desulfovibrio was significantly positively correlated with both body length and tail length (*p* < 0.05); Norank_Muribaculaceae was significantly negatively correlated with the cranial height of long-tailed dwarf hamsters (*p* < 0.01); and Candidatus_Saccharimonas was significantly negatively correlated with the cranial base length, upper tooth length (left), and lower tooth row length (left) of the hamsters (*p* < 0.05). As shown in Figure 7B, Norank_Muribaculaceae and Lactobacillus had the most significant effects on the physical characteristics of long-tailed dwarf hamsters. Norank_Muribaculaceae exhibited negative relationships with cranial height, neurocranium width, body length, as well as upper tooth row length (left; *p* < 0.05), and positive correlations with cranial length, cranial basal length, and lower tooth row length (left; *p* < 0.05). Conversely, Lactobacillus exhibited negative correlations with cranial height, cranial length, cranial basal length, and lower tooth row length (left; *p* < 0.05), but positive correlations with neurocranium width, body length, and upper tooth row length (left; *p* < 0.05).

### 3.5 Functional analysis of gut microbiota of long-tailed dwarf hamsters at various elevations

**Figure 8:**
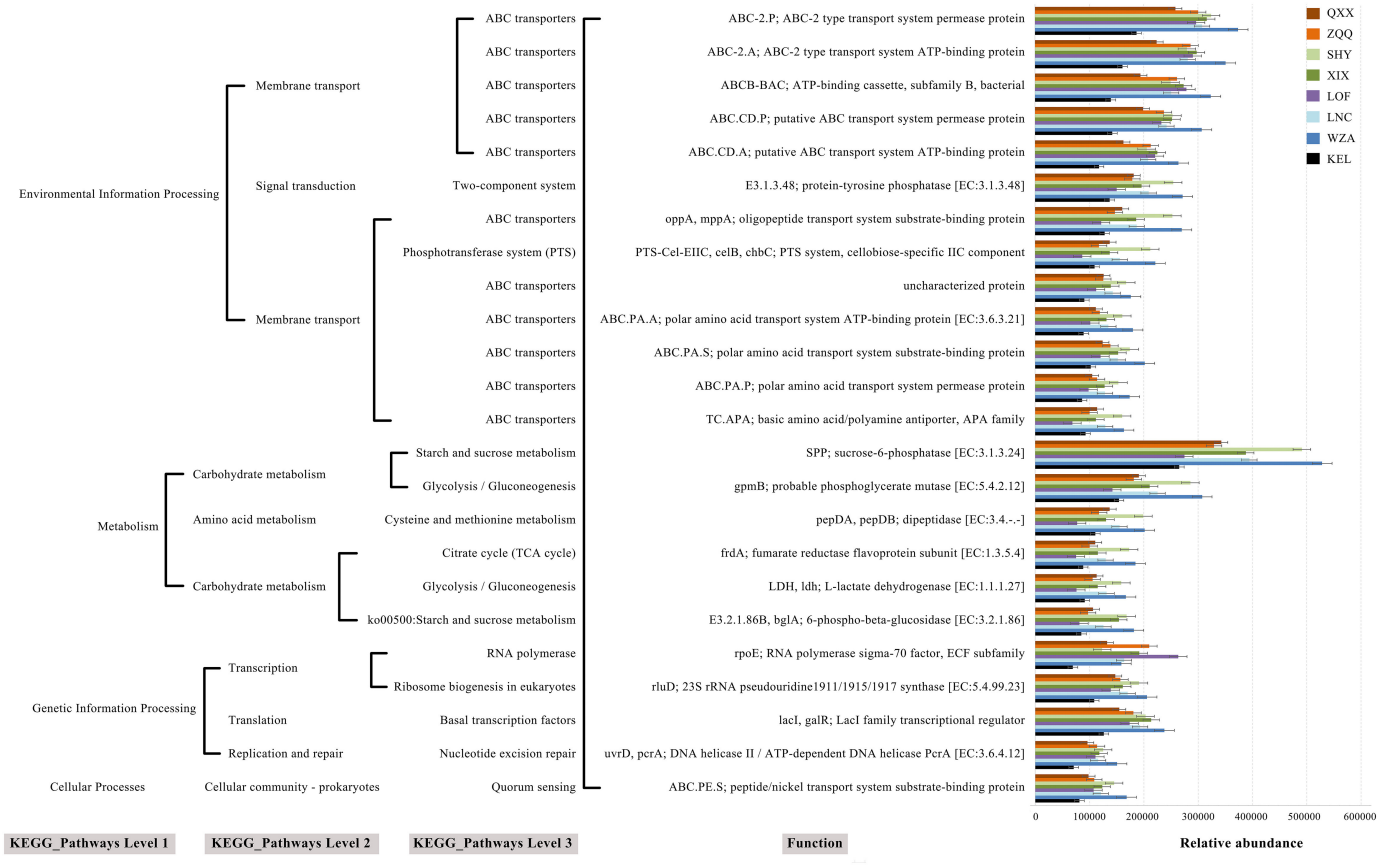
The KEGG pathway at eight altitudes was significantly different in the functional prediction of rumen microbiota.

The metabolic role of gut microbiota in long-tailed dwarf hamsters at eight altitudes was predicted using PICRUSt2 (Figure 8). We acquired the Pathway and Module labeling information for the sequence based on the correlation between KO and Pathway and Module. The findings showed that cellular processes, metabolism, genetic information processing, and environmental information processing were all included in the modified metabolic pathways. Among these metabolic processes, which include vital metabolic pathways like glycolysis/gluconeogenesis and cysteine and methionine metabolism, the abundance rose with altitude but KEL animals fell in comparison to other places. Interestingly, across all eight altitudes, the relative abundance of ABC transporters was highest.

### 3.6 Co-occurrence network of gut microbes in long-tailed dwarf hamsters at varying elevations

**Figure 9:**
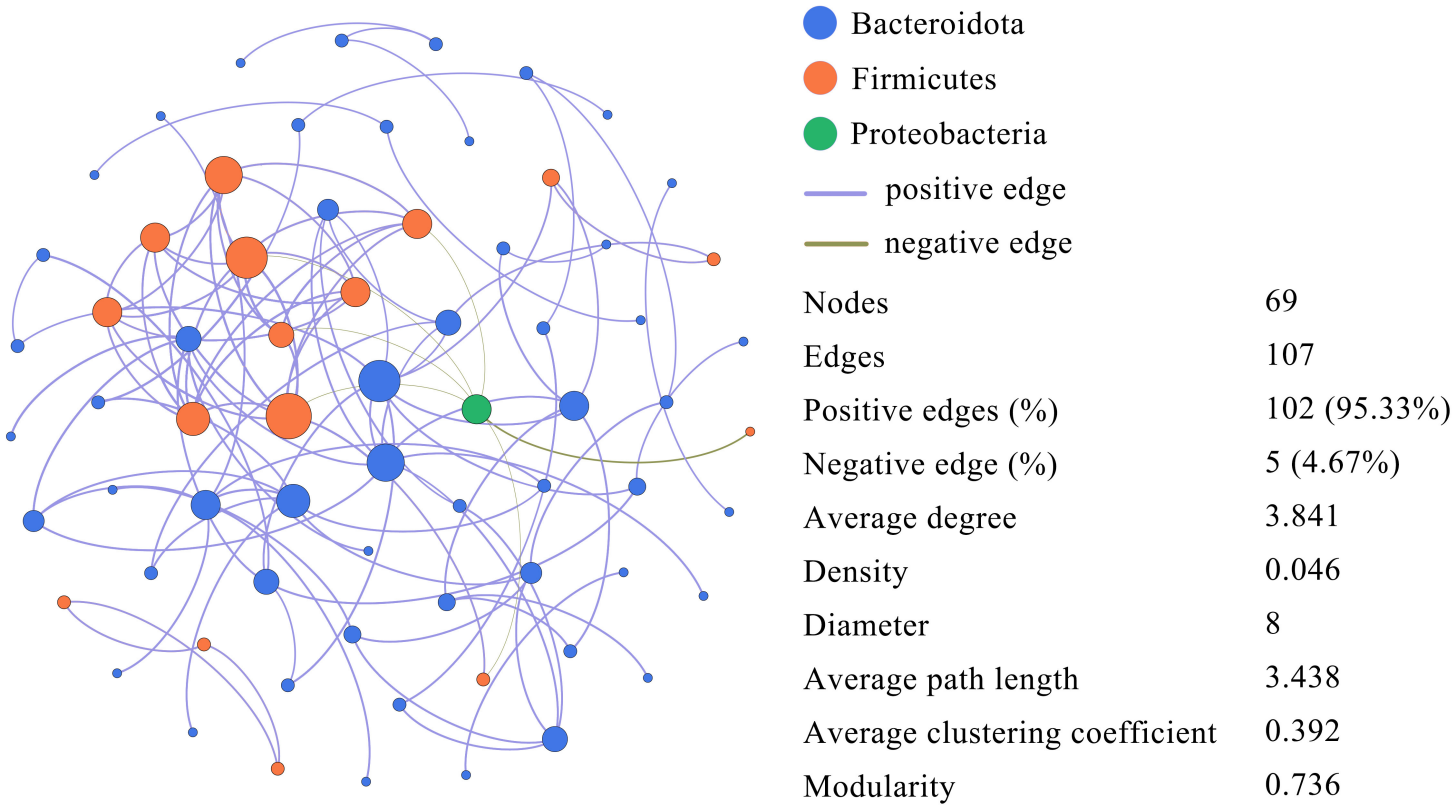
Dominant OTU co-occurrence networks in *Cricetulus longicaudatus*.

Using microbial interactions (OTU with *r* > 0.6, *p* < 0.05, and abundance > 0.001 for all samples), Figure 9 shows the correlation of dominant OTUs in the gut microbiota of long-tailed dwarf hamsters at various elevations. Microorganisms with *r* > 0.6 and *p* < 0.05 have a Spearman's rank correlation, which shows that they are positively correlated (purple edges) and negatively correlated (green edges). The network comprises 107 edges and 69 nodes, with an average of 1.551 edges per node. The modularity index (MD) is 0.736, the average path length (APL) is 3.438, and the average clustering coefficient (ACC) is 0.392. A value >0.4 indicates a modular structure in the network. We identified three bacterial phyla from the network's nodes, with Bacteroidetes and Firmicutes accounting for 98.55% of all nodes and exhibiting extensive spread.

## 4 Discussion

For more than 80 million years, the gut microbiota has co-evolved with its host, and because of its metabolic capacities, it has helped animals' ecological dispersal range (Zhang K. F. et al., 2023). Likewise, gut microbiota abundance and composition are influenced by the host's habitat environment. In this study, long-tailed dwarf hamsters' gut microbiomes showed consistent and notable changes as altitude increased. Long-tailed dwarf hamsters' guts continued to be dominated by Firmicutes and Bacteroidetes at the phylum level, which is in line with earlier research (Cao et al., 2024). Previous research has shown that variations in seasonal nutrition are linked to changes in the gut microbiota composition and *F*/*B* ratios in wild animals (Springer et al., 2017; Baniel et al., 2021). According to a study on wild geladas (*Theropithecus gelada*), their summer diet has more fiber (and lignin) than their winter diet (Baniel et al., 2021). Nonetheless, the observed decline in the *F*/*B* ratio among long-tailed dwarf hamsters that were caught in the summer suggests that these animals have species-specific adaptations to altitude changes. Firmicutes play a crucial role in aiding food absorption and energy conversion, as several studies have shown (Turnbaugh et al., 2006), while Bacteroidetes have roles in proteolysis and the breakdown of carbohydrates (Thomas et al., 2011). Additionally, as altitude increased, so did Proteobacteria, Desulfobacterota, and Spirochaetota. Lignin, cellulose, and metabolic processes may be catabolized by Proteobacteria, Desulfobacterota, and Spirochaetota (Sun et al., 2019; Gorbunov et al., 2022; Salgado et al., 2024). There were more Actinobacteriota and Patescibacteria at higher elevations than at lower elevations, which suggests that they may be involved in the metabolism and absorption of nutrients (Zhu et al., 2024). These findings are consistent with earlier research on yaks and mice (Fan et al., 2020; Chevalier et al., 2015) and suggest that long-tailed dwarf hamsters living in lower elevation areas may have improved food absorption, energy conversion, and adaptation. In the present study, the two most common genera of gut microbiota at the genus level were Lactobacillus and unclassified Lactobacillaceae. Notably, other studies have shown that Lactobacillus can boost animals' immunity at high elevations (Hao et al., 2024). Furthermore, Tang et al. (2023), found norank_Muribaculaceae at high elevations and closely linked it to a diet high in fat. A study by Tang et al. (2023) found that, contrary to what other studies had found, the number of Lactobacillus bacteria increased with height, while the number of norank_Muribaculaceae bacteria decreased with height. The study of gut microbiota and environmental factors at the same time also found a strong negative correlation between norank_Muribaculaceae and wind speed and altitude, but a strong positive correlation between norank_Muribaculaceae and temperature. We discovered that altitude had a greater impact on the abundance of norank_Muribaculaceae in long-tailed hamsters because there is less high-fat food available at higher elevations. However, there was no discernible change in the summertime temperatures. Notably, norank_Muribaculaceae also significantly influenced the phenotypic alterations of long-tailed dwarf hamsters. Gut microbiota exhibited a favorable cooperative relationship that efficiently assisted their adaptation to a variety of adverse conditions, according to the co-occurrence network analysis (Li et al., 2019). According to these findings, long-tailed dwarf hamsters may be able to regulate these genera to thrive at high elevations by utilizing their species-specific adaptations.

According to the alpha analysis of the gut microbiota in long-tailed dwarf hamsters, the KEL individuals' gut microbiota diversity index was noticeably lower than that of the other seven regions. The microbiological evenness of these eight locations did not, however, differ much, according to Shannon diversity analysis. The relatively high elevation and limited food supply in KEL may help explain this finding. This is because long-tailed dwarf hamsters that live there have the least variety of microbes in their guts (Zhang Z. Y. et al., 2023). In contrast to earlier findings (Yan et al., 2022), the weighted unifrac PCoA and hierarchical clustering analysis results in this study showed significant differences between KEL animals and the other regions, indicating that altitude affects the gut microbiota structure of long-tailed dwarf hamsters. The findings of the Venn diagram showed that WZA had the lowest abundance and ZQQ and LOF the maximum number of taxa, suggesting that changes in altitude can affect the microbial diversity of long-tailed hamsters. According to research, beneficial bifidobacteria numbers sharply decline at high elevations (Kleessen et al., 2005). In the present study, Bifidobacterium was significantly more common in the other seven regions in the present study than it was in the high-altitude KEL region. Additionally, the KEL individuals had a lot more Providencia, unidentified Lactobacillaceae, Myroides, and Acinetobacter. While unclassified Lactobacilllaceae has been found as a helpful bacterium (Tian et al., 2019), previous investigations have shown that Providencia and Myroides are pathogenic (O'Hara et al., 2000; Schröttner et al., 2014).

A healthy gut microbiota has been shown to have a positive effect on host metabolic homeostasis, and environmental influences can also affect the gut microbiota's composition and function (Dey et al., 2015). According to the present results, height and wind_speed significantly correlated positively with Providencia, whereas humidity significantly correlated negatively with Providencia. Furthermore, we observed a complex correlation between Providencia and phenotypic changes in long-tailed dwarf hamsters. This suggests that long-tailed hamsters change the bacteria that live in their guts to deal with the problems and potentially harmful bacteria that they face at high elevations. This shows how altitude and gut microbiota can affect how an animal survives. One of the most important questions in biology is the study of large-scale, systematic body size evolution. Understanding animal life history requires being able to recognize changes in animal body size in response to environmental variables such as temperature, altitude, and latitude (Lou et al., 2012). Animals will change the shape of their bodies and skulls to adapt to their surroundings (Yan et al., 2022). According to Bergmann's law (Freckleton et al., 2003), which states that body weight increases with altitude and decreases with temperature, the results of this study show a positive association between altitude and body weight in long-tailed dwarf hamsters. These findings align with earlier research on tiny rodents (Yom-Tov and Yom-Tov, 2004; Roycroft et al., 2020; Cui et al., 2022). Variations in body size can be reflected in the size of the skull, which serves as a constancy organ in animals. *Apodemus draco* and *Niviventer confucianus* had different cranial base lengths in different parts of the world, so Bergmann's rule wasn't always useful for them (Huang et al., 2012). This study discovered that when long-tailed dwarf hamsters adapted to a changing altitude environment, their cranial morphology increased as altitude climbed. Our research shows that long-tailed dwarf hamsters' cranial morphology increases with altitude, which is in line with a prior study on *E. cansus, E. baileyi, E. rufescens, E. smithii*, and *E. fontanierii* (Kang et al., 2021). This means that changes in elevation may have an effect on the availability of resources and the way that different species compete with each other, which may then have an effect on the shape of skulls. According to Allen's law, which is an extension of Bergmann's rule, endotherms' appendage dimensions (such as theirtailsl, limbs, and outer ears) have a positive association with latitude and a negative correlation with temperature and elevation (Allen, 1877). In this study, the long-tailed dwarf hamsters' tail length, left forelimb length, and left hindlimb length showed a significant positive connection with altitude. Furthermore, environmental variables including humidity and windspeed and also had an impact on the phenotypic traits of long-tailed dwarf hamsters.

According to this study, the long-tailed hamster's gut microbiota is also crucial in determining its phenotypic changes, and we found a favorable relationship between Providencia abundance and these modifications. It has been demonstrated that Providencia may be a gastrointestinal pathogen (O'Hara et al., 2000). Its impact on animal phenotypes is still unknown, though, and requires more research to confirm.

We used PICRUSt2 in this study to predict the metabolic activity of microbial populations in the guts of long-tailed dwarf hamsters. The findings showed that the long-tailed dwarf hamster's gut microbiome's metabolic activities changed a lot with altitude. As altitude rose, metabolic processes related to carbohydrates increased (Martin et al., 2010). This implies that long-tailed dwarf hamsters' gut microbiomes has improved energy production capacities with altitude, which promotes optimal nutrient utilization and effective breakdown of indigestible plant components like cellulose. This suggests that the long-tailed hamster's gut microbiota generates a substantial amount of energy as altitude rises, helping the host maximize food usage and digest indigestible plant materials like cellulose (Fuchs, 2011). It's interesting that ABC transporters directly help make ATP and are the most common way that membranes move things around (Hamana et al., 2012). According to this study, altitude increased the expression level of ABC transporters. Genes involved in replication and repair are necessary to counteract the biomolecular deficiencies caused by high-altitude circumstances, which can cause damage to proteins and DNA. Thus, this pathway might aid in the adaptation of long-tailed dwarf hamsters to high elevations. However, at a specific altitude (KEL region), these metabolic processes were reduced, indicating that the KEL hamster modified its survival strategy to lower energy expenditure in order to deal with the high altitude difficulty. It should be highlighted, nonetheless, that our results might not fully represent how the gut microbiota functions in long-tailed dwarf hamsters; hence, more research is required.

## 5 Conclusion

For the first time, we compared the gut microbiota of long-tailed dwarf hamsters in Shanxi Province at various elevations. Results showed that Firmicutes and Bacteroidetes were the most common phyla, and Lactobacillus was the most common genus. We found that gut microbiota varied by location and that altitude had impacted this diversity. People living in the high-altitude regions exhibited less β diversity in their gut microbiota than those living in low-altitude regions. Additionally, long-tailed dwarf hamsters' skeleton and skull indices varied with elevation. According to the study's findings, long-tailed dwarf hamsters' body sizes follow Bergmann's law. Additionally, there was a strong link between Providencia and body size. Finally, functional study of the gut microbiota revealed changes in metabolic function that were dependent on altitude, and collinear network analysis showed how the gut bacteria interacted with one another. Our results showed clear differences in the makeup of the gut microbiota between long-tailed dwarf hamsters at different elevations, with altitude being the main factor affecting the microbial structure and metabolic activity of these creatures. The study's conclusions shed important light on how the gut microbiota helps wild mice adapt to different elevations, which advances our knowledge of their physiological and ecological reactions.

## Data Availability

16s rDNA data have been uploaded to the European Nucleotide Archive database (http://www.ebi.ac.uk/ena). The data entry number is PRJEB82708. Physiological data upload to Figshare database (https://figshare.com/), the data acquisition of doi number is: 10.6084/m9.figshare.27886629.v1.
